# A protocol for custom CRISPR Cas9 donor vector construction to truncate genes in mammalian cells using pcDNA3 backbone

**DOI:** 10.1186/s12867-018-0105-8

**Published:** 2018-03-14

**Authors:** Neftali Vazquez, Lilia Sanchez, Rebecca Marks, Eduardo Martinez, Victor Fanniel, Alma Lopez, Andrea Salinas, Itzel Flores, Jesse Hirschmann, Robert Gilkerson, Erin Schuenzel, Robert Dearth, Reginald Halaby, Wendy Innis-Whitehouse, Megan Keniry

**Affiliations:** 1 Department of Biology, University of Texas-Rio Grande Valley, 1201 W. University Dr., Edinburg, TX 78539 USA; 2 Department of Biology, Montclair State University, 1 Normal Ave., Montclair, NJ 07043 USA; 3 School of Medicine, University of Texas-Rio Grande Valley, 1201 W. University Dr., Edinburg, TX 78539 USA

**Keywords:** CRISPR Cas9, Mammalian cell lines, Custom donor vector design and construction

## Abstract

**Background:**

Clustered regularly interspaced short palindromic repeat (CRISPR) RNA-guided adaptive immune systems are found in prokaryotes to defend cells from foreign DNA. CRISPR Cas9 systems have been modified and employed as genome editing tools in wide ranging organisms. Here, we provide a detailed protocol to truncate genes in mammalian cells using CRISPR Cas9 editing. We describe custom donor vector construction using Gibson assembly with the commonly utilized pcDNA3 vector as the backbone.

**Results:**

We describe a step-by-step method to truncate genes of interest in mammalian cell lines using custom-made donor vectors. Our method employs 2 guide RNAs, mutant Cas9D10A nickase (Cas9 = CRISPR associated sequence 9), and a custom-made donor vector for homologous recombination to precisely truncate a gene of interest with a selectable neomycin resistance cassette (*NPTII: Neomycin Phosphotransferase II*). We provide a detailed protocol on how to design and construct a custom donor vector using Gibson assembly (and the commonly utilized pcDNA3 vector as the backbone) allowing researchers to obtain *specific* gene modifications of interest (gene truncation, gene deletion, epitope tagging or knock-in mutation). Selection of mutants in mammalian cell lines with G418 (Geneticin) combined with several screening methods: western blot analysis, polymerase chain reaction, and Sanger sequencing resulted in streamlined mutant isolation. Proof of principle experiments were done in several mammalian cell lines.

**Conclusions:**

Here we describe a detailed protocol to employ CRISPR Cas9 genome editing to truncate genes of interest using the commonly employed expression vector pcDNA3 as the backbone for the donor vector. Providing a detailed protocol for custom donor vector design and construction will enable researchers to develop unique genome editing tools. To date, detailed protocols for CRISPR Cas9 custom donor vector construction are limited (Lee et al. in Sci Rep 5:8572, 2015; Ma et al. in Sci Rep 4:4489, 2014). Custom donor vectors are commercially available, but can be expensive. Our goal is to share this protocol to aid researchers in performing genetic investigations that require custom donor vectors for specialized applications (specific gene truncations, knock-in mutations, and epitope tagging applications).

**Electronic supplementary material:**

The online version of this article (10.1186/s12867-018-0105-8) contains supplementary material, which is available to authorized users.

## Background

A significant proportion of bacteria and archaea (roughly 40 and 90% respectively) employ [1, 2] CRISPR Cas9 mechanisms as an adaptive immunological response against virus and plasmid foreign DNA [3–10]. Researchers have exploited the CRISPR Cas9 molecular machinery to target genes in numerous organisms such as yeast, flies, worms, and mammals leading to groundbreaking discoveries [11–14]. Although other approaches have been utilized for genome editing for decades, CRISPR Cas9 technology has reshaped genetic engineering by providing a quick and facile tool, greatly accelerating research [13, 14].

Endogenous CRISPR Cas9 (and related) systems serve as an acquired immunological response [3–5, 15–18]. Invading DNA (from plasmids and viruses) becomes incorporated into the *CRISPR* locus of the prokaryotic genome. *CRISPR* loci typically have noncontiguous direct repeats and spacers that contain the invading DNA sequences [19]. Transcription of the *CRISPR* locus produces a pre-crRNA (crRNA = CRISPR RNA) that base pairs with a trans-activating-crRNA (tracrRNA, also encoded by CRISPR system), leading to processing and incorporation into a Cas9-containing complex [20–22]. Many prokaryotes harbor specific endonucleases such as Cas9 that contain two domains: RuvC-like [an endonuclease domain named for an *Escherichia coli* (*E. Coli*) protein involved in DNA repair] and HNH (an endonuclease domain named for characteristic histidine and asparagine residues) to cleave foreign DNA [15]. Hybridized crRNA/tracrRNA serves as a guide for Cas9 to cleave foreign DNA in a sequence-specific manner. With heterologous CRISPR Cas9 systems such as those utilized in human cells, a chimeric guide RNA (gRNA) is employed to target specific sequences [23]. The gRNA contains a fusion between tracrRNA and crRNA that enables specific targeting of Cas9 to a gene of interest [24].

Employing CRISPR Cas9 technology as a gene editing tool is a recent development in the field of molecular biology [14, 24]. This tool has already had a transformative impact on research, allowing for the quick identification of mutations in wide-ranging experimental settings [23]. However, it has become increasingly evident that utilization of CRISPR Cas9 can lead to off-target effects [19, 25, 26]. CRISPR Cas9 can tolerate base pair mismatches between the gRNA and target sequences [25, 27, 28]. CRISPR technology utilizes the host DNA repair machinery to resolve DNA lesions, leading to the isolation of mutations [29]. One issue that we encountered when trying to mutate genes in cancer cell lines, widely reported by others, was that mutation frequencies vary widely depending on the methodology employed, the locus being mutated and screening methods [17, 29]. Chiang et al. observed mutation efficiencies without selection by green fluorescent protein (GFP)-based cell sorting of 1–4% in HAP1 (near diploid chronic myelogenous leukemia) and 2–22% in U2OS (human bone osteosarcoma epithelial) cell lines [28, 30–32]. In these settings with low mutation frequencies, methods that employ selection (such as neomycin resistance) may be necessary to obtain enough mutants for study in a cost effective manner. Here, we describe a detailed protocol to construct a custom donor vector (using the pcDNA3 vector as the backbone) in order to truncate the gene of interest. We describe a streamlined screening process to isolate and validate mutants in settings with low mutation frequencies.

## Methods

**Fig. 1:**
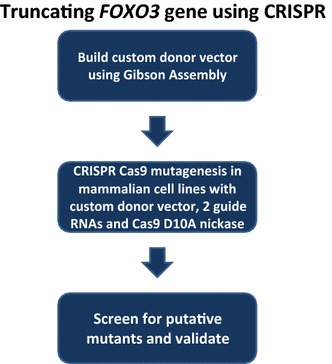
Flow chart for CRISPR Cas9 mutagenesis using a custom donor vector. A flow chart for CRISPR mutagenesis with custom donor vector is depicted

**Fig. 2:**
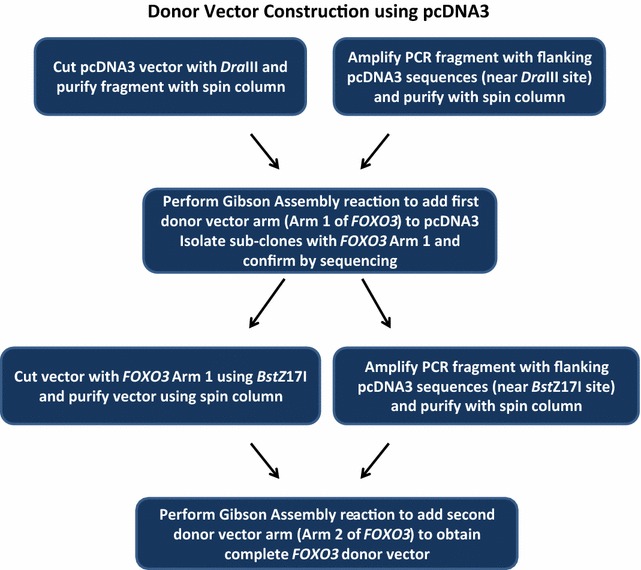
Construction of *FOXO3* donor vector. A fragment of *FOXO3* (Arm 1) was inserted into the pcDNA3 vector proximal to the *Dra*III restriction site by using Gibson assembly. This intermediate vector was called *FOXO3* Arm1 and was utilized to make the final *FOXO3* donor vector. Another fragment of *FOXO3* (Arm 2) was inserted into the *FOXO3* Arm1 vector (by Gibson assembly) to obtain the final *FOXO3* donor vector. This vector was confirmed by Sanger sequencing and employed in CRISPR Cas9 mutagenesis reactions

**Fig. 3:**
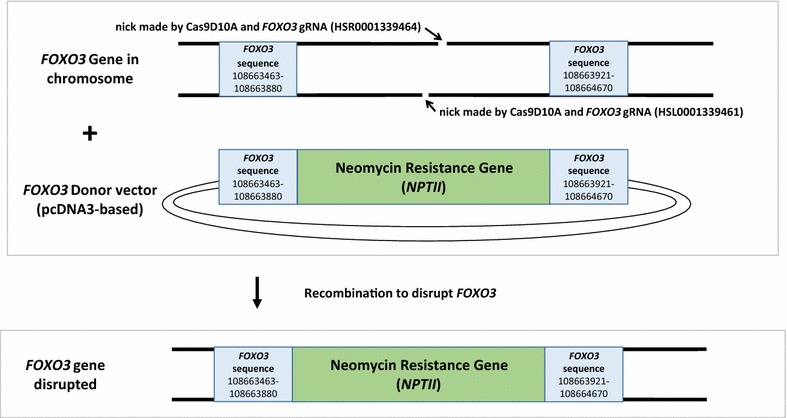
Schematic of *FOXO3* gene disruption with neomycin resistance cassette (*NPTII*). Guide RNAs were employed to nick *FOXO3* gene in mammalian cell lines. The lesions were resolved by recombination with a donor vector that contained a neomycin resistance gene (*NPTII*) flanked by *FOXO3* sequences (that were proximal to the CRISPR Cas9-generated nicked strands of *FOXO3* in the chromosome)

This protocol can be employed to specifically truncate genes of interest using a neomycin resistance cassette (*NPTII*, enabling selection of mutants with G418) in mammalian cell lines. Figure 1 shows the basic steps to truncate the gene of interest. First, a custom donor vector needs to be designed and constructed. Next, CRISPR Cas9 mutagenesis needs to be performed. Last, mutants must be isolated and validated. This protocol addresses multiple barriers found with employing CRISPR Cas9 to mutate genes. First, we needed to design a custom donor vector in order to obtain truncation mutants for our research. Although we found many protocols for CRISPR mutagenesis, we found a lack of published protocols that described custom donor vector construction in detail and purchasing a custom donor vector can be expensive [1, 2]. Secondly, we sought to minimize off-target effects [25, 28, 30–32]. Finally, we wanted to be able to quickly isolate mutants for a gene of interest, even if the mutation frequency was low. Many cancer cell lines such as U87MGs have deficient homologous recombination repair, making CRISPR mutagenesis inefficient [33, 34]. Our approach utilized transiently transfected Cas9D10A nickase, two gRNAs, and a donor vector to disrupt *FOXO3* gene coding sequence with a neomycin resistance gene. The custom donor vector was built using two, separate Gibson assembly cloning steps with the pcDNA3 vector (Figs. 2, 3).

## Construction of *FOXO3* donor vector using Gibson assembly

Gibson assembly is an extremely efficient method to obtain insertions into a plasmid vector of interest [35]. The *FOXO3* donor vector was prepared using a two-step Gibson assembly-based cloning procedure. The complete sequence of the *FOXO3* donor vector can be found in Additional file 1: Figure S1. Figures 2, 3 depict the steps employed to prepare a custom *FOXO3* donor vector. First, a 418 base pair fragment from the *FOXO3* gene (named *FOXO3* Arm 1) was inserted into the pcDNA3 vector just upstream of the neomycin resistance cassette (*NPTII*), producing an intermediate vector. In the second sub-cloning step, (shown in Figs. 2, 3) a 750 base pair *FOXO3* fragment (Arm 2) was inserted into the donor vector.

For optimal results, *FOXO3* donor vector sequences were selected for (1) proximity to genomic nick sites and (2) sufficient sequence length to permit efficient recombination. It is important to note that there are two nick sites for *FOXO3* in the genome when using 2 gRNAs and the Cas9D10A nickase. Therefore, the upstream *FOXO3* fragment (Arm 1) in the donor vector and downstream *FOXO3* fragment (Arm 2) need to be in regions that are in positions amenable to recombination with the two genomic CRISPR nick sites. The *FOXO3* donor vector fragments should be within 20 bases of the nick sites and should have at least a few hundred base pairs to promote recombination between the donor vector and the chromosome [26]. In our design, the upstream sequence used in the donor vector for *FOXO3* Arm 1 contained 418 chromosomal *FOXO3* base pairs; these sequences ended seven bases upstream of the gRNA targeting site in the genome, allowing for 418 base pairs of homology between the donor vector and genome for recombination-mediated repair just before the nick site in the genome. The distance between the bottom strand and the top strand nick sites made by CRISPR Cas9 D10A was 51 bases. The donor vector sequences in *FOXO3* Arm 1 and *FOXO3* Arm 2 were non-overlapping. The downstream fragment in the donor vector contained 750 bases that were homologous to *FOXO3* chromosomal DNA (that extended beyond the second nick site further downstream into the gene) in order to promote recombination; the second nick site was 12 bases from the start of the *FOXO3* Arm 2.

## Step-by-step Gibson assembly reactions for the *FOXO3* donor vector

Gibson assembly reactions were performed to insert two *FOXO3* fragments into the pcDNA3 vector at positions that were on either side of neomycin resistance cassette (*NPTII* gene). For the Gibson assembly reaction, there needed to be identical sequences on the ends of each piece of DNA that would be physically joined. Therefore, the ends of each PCR product needed to be identical to the piece of pcDNA3 vector to which it would be fused. We added pcDNA3 vector sequences to the 5′ ends of PCR primers utilized to amplify *FOXO3* fragments. Therefore, *FOXO3* gene fragments (PCR products) had pcDNA3 sequences on the ends that corresponded to upstream and downstream sequences of the utilized restriction sites (*Dra*III for Arm1 and *Bst*Z17I for Arm2) in pcDNA3.

### Addition of *FOXO3* Arm 1 to donor vector

The pcDNA3 vector was cleaved with *Dra*III (restriction enzyme from NEB, Ipswich, MA) for 2 h at 37 °C. The restriction digest included 1 µg of the pcDNA3 vector, 4 μL of 10× NEB Cut Smart buffer, 3 μL of *Dra*III restriction enzyme and 27 μL of water. After this, 1 μL of calf intestine phosphatase (CIP) was added to the reaction (from NEB, Ipswich, MA) and incubated for 1 more hour at 37 °C. The digested and phosphatased vector was column purified using Qiagen PCR purification system (Hilden, Germany). DNA was eluted with sterile water and quantified using a Nanodrop spectrophotometer.

In order to obtain the *FOXO3* Arm 1 for sub-cloning, a PCR product was prepared that had a fragment of the *FOXO3* gene (adjacent to the CRISPR nick sites in the genome) with sequences on the end of each primer that were identical to the pCDNA3 vector next to the *Dra*III site; see Table 1 for PCR primer sequences. The *FOXO3* fragment was within 20 bases of the nick in the genome for recombination. This fragment was 418 base pairs in length, allowing a significant amount of homology to promote recombination between the donor vector and the nicked chromosome. The *FOXO3* fragment was prepared using Phusion high fidelity polymerase (Thermo-Fisher, Waltham, MA). The *FOXO3* Arm 1 PCR product was cleaved with *Dpn*I for 3 h to remove plasmid DNA template (used as template for PCR reaction); 2 μL of *Dpn*I (NEB, Ipswich, MA) were added to unpurified *FOXO3* PCR product (still containing nucleotides, polymerase, etc.) and samples were incubated at 37 °C for 2 h. After *Dpn*I digestion, the PCR product was column purified using Qiagen PCR purification system (Hilden, Germany) and eluted with sterile water. The purified PCR product was quantified with a Nanodrop spectrophotometer.

**Table 1:** PCR Primers utilized to amplify *FOXO3* gene fragments for Donor Vector Gibson Assembly Reactions

Primer	Sequence^a^
*FOXO3* ARM 1 F	5′-**GTGCTTTACGGCACCTCGACCCC***CCGGCACAACCTGTCACTGC-3′*
*FOXO3* ARM 1 R	5′-**CCGTCTATCAGGGCGATGGCC***GCTGTAGAGCATGGGCGAGAG-3′*
*FOXO3* ARM 2 F	5′-**CAAATAAAGCATTTTTTTCACT***CGGTGGAACTGCCACGGCTG-3′*
*FOXO3* ARM 2 R	5′-**GAGTTTGGACAAACCACAACTA***GGTCCAAGTCGCTGGGGAAC-3′*

^a^Nucleotides in bold corresponds to pcDNA3 vector sequences. Sequences in italics were to amplify the indicated *FOXO3* arm

Gibson assembly reactions were performed using 10 ng of vector (cut, phosphatased and column purified pcDNA3) with 80 ng of insert (*FOXO3* fragment); the DNA reactants comprised a volume of 10 μL initially. To the DNA reactants, 10 μL of NEB Gibson assembly mix was added for a final volume of 20 μL (NEB, Ipswich, MA). These reactions were incubated at 50 °C for 1 h and were then transformed into chemically competent bacterial cells (5-alpha competent *E. coli*, NEB, Ipswich, MA) as directed by the NEB Gibson assembly kit. Transformed bacterial cells were plated in dilutions (1:10, 1:100 and 1:1000) to obtain single colonies given the high efficiency of the Gibson assembly reactions. Single colonies were screened by restriction digest and confirmed by Sanger sequencing. The vector obtained from this was called *FOXO3* Arm 1 vector.

### Addition of *FOXO3* Arm 2 to donor vector

The second arm for the *FOXO3* donor vector was prepared in a similar manner to Arm 1. The intermediate vector (with *FOXO3* Arm 1) was cut with *Bst*Z17I, which is on the other side of the neomycin resistance gene in the pcDNA3 plasmid compared to *FOXO3* Arm 1. The cleaved vector was treated with CIP (1 μL CIP, NEB, Ipswich, MA) for 1 h and subsequently column purified. *FOXO3* Arm 2 was amplified with the primer pair specified in Table 1, producing a product that had sequences on each end that were identical to the sequences proximal to the *Bst*Z17I site in the intermediate *FOXO3* Arm 1 vector. Gibson assembly reactions were performed (as previously described to sub-clone Arm 1 of *FOXO3*) to obtain the final *FOXO3* donor vector Figs. 2, 3. Transformed bacterial cells were plated in dilutions (1:10, 1:100 and 1:1000) to obtain single colonies given the extremely high efficiency of the Gibson assembly reactions. The complete *FOXO3* donor vector was confirmed by restriction fragment analysis and Sanger sequencing. The complete sequence of the *FOXO3* donor vector can be found in Additional file 1: Figure S1.

## CRISPR Cas9 mutagenesis to truncate the *FOXO3* gene in mammalian cells

### Transient transfections to obtain *FOXO3* truncation mutants

Transient transfections were performed using the glioblastoma cell line U87MG, breast cancer cell line BT549, or human kidney cell line HEK 293, which were obtained from American Type Culture Collection (ATCC, Manassas, VA) and propagated under standard conditions [37 °C with 5% CO_2_ in media specified by ATCC supplemented with 10% FBS (fetal bovine serum) and 5% penicillin/streptomycin (pen/strep)]. Media employed to grow U87MG cells was MEM, BT549 was RPMI and HEK 293 was DMEM. One million cells of each cell line was transfected using LONZA (nucleofection kit V, program P-20 for U87MG and BT549 and program X-001 for HEK 293) and were allowed to recover for 2 days in 10 cm dishes. In each transfection, 250 ng of each guide RNA (gRNA) vector (Table 2) were added as well as 250 ng of the vector enabling Cas9D10A expression (CRISPR Cas9D10A-GFP Nickase, catalog: CAS9D10AGFPP, Sigma, St. Louis, MO). Guide RNA vectors were obtained from Sigma (St. Louis, MO) and were utilized to make DNA nicks in both chromosomal *FOXO3* DNA strands (Figs. 2, 3). The *FOXO3*-containing portions of the gRNAs are detailed in Table 2. A negative control gRNA (CRISPR Universal Negative Control 1, catalog: CRISPR06, Sigma, St. Louis MO) was utilized for control experiments (500 ng per transfection to have the same amount of DNA as mutagenesis samples). One microgram of *FOXO3* donor vector was utilized in each transfection.

**Table 2:** Guide RNA sequences

Gene	I.D. for construct	Sequence
*FOXO3*	HSL0001339461	CTTACTGAAGGTGACAGGCTGG
*FOXO3*	HSR0001339464	CACGGCTGACTGATATGGCAGG

It is important to note that the nicks directed by the gRNAs are staggered on the chromosome (about 40 base pairs apart). These gRNAs are used in concert with the Cas9D10A nickase (CRISPR Cas9D10A-GFP Nickase, catalog: CAS9D10AGFPP, Sigma, St. Louis, MO) to make nicks in a gene of interest [26, 28, 30–32]. It has been shown that Cas9D10A allows for > 100-fold increased specificity for genomic editing (between 200-fold and 1500-fold based on deep sequencing experiments) [26].

For each mutagenesis, cells that survived the transfection (after 2 days of recovery) were incubated in 0.25% trypsin for 3 min and were placed into 10 mL of MEM (contained 10% FBS and 5% Pen/Strep). Ten plates of diluted cells (approximately 100,000 cells per 10 cm dish) were prepared from this mixture. G418 (0.5 mg/mL final concentration for U87MG and BT549 and 1.5 mg/mL G418 for HEK 293 cells) was added to each 10 cm plate 1 day after plating. Single clones were isolated from these selected dishes 4 weeks later using cloning cylinders. 2–10 single colonies were obtained from each 10 cm plate. To clone a colony, the plate was washed with 2 mL 0.25% trypsin and aspirated. Cloning cylinders (Fisher: 0955221) were placed onto the 10 cm plate using sterile forceps and vaseline (to make the cylinder stick to the plate). 200 μL of 0.25% trypsin was added to each cylinder and incubated for 5 min. The 200 μL of trypsin was pipetted up and down ten times and then plated into 2 mL of fresh media in a well of a six well plate.

## Results

### Western blot analysis with putative FOXO3 mutants

**Fig. 4:**
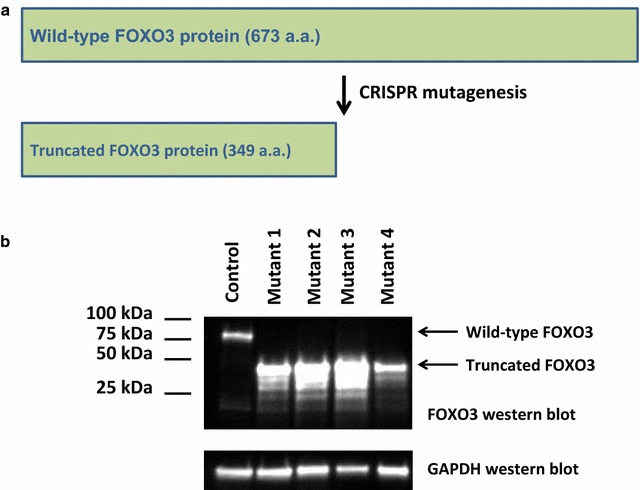
FOXO3 truncation mutant proteins retain the DNA binding domain. **a** Disruption of the *FOXO3* gene as described in “Methods” led to a truncated protein that was 349 amino acids in length. This mutant protein retained the FOXO3 DNA binding domain, but lacked the transactivation domain. **b** Total protein lysates prepared from *FOXO3* mutant-containing cells and control cells were examined by western blot analysis; employed antibodies are indicated. Wild-type FOXO3 was approximately 80 kDa, whereas, mutant FOXO3 protein was approximately 45 kDa. Out of 77 putative mutants screened, only four were homozygous mutant (confirmed by Sanger sequencing)

Proof-of-principle studies were done to determine mutation frequencies using our described protocol in several mammalian cell lines: U87MG, BT549 and HEK 293. Figure 4 depicts the encoded FOXO3 truncation mutant protein. FOXO3 is an extensively characterized transcriptional activator that impacts metabolism, the cell cycle, apoptosis and cell fate [36–38]. Disruption of the *FOXO3* gene led to the production of a truncated mutant protein that contained the first 349 amino acids of FOXO3 (including the DNA binding domain); most of the transactivation domain was deleted. CRISPR Cas9 mutagenesis followed by selection with neomycin with U87MG cells allowed our group to isolate 77 putative mutants in one trial and 50 putative mutants in another. We confirmed the truncation mutants in several ways. Western blot analyses were performed as described previously [39]. Expression of FOXO3 was assessed by western blot analysis. Total protein was obtained from U87MG cells by rinsing out the 6 well plate wells with 1XPBS followed by directed lysis in 2× sample buffer (125 mM Tris–HCL at pH 6.8, 2% sodium dodecyl sulfate (SDS), 10% 2-mercaptoethanol, 20% glycerol, 0.05% bromophenol blue, 8 M urea); 2× sample buffer was added to each well and scraped. The lysate was collected from each well, placed into 1.5 mL microcentrifuge tubes and heated for 10 min at 90 °C in a dry-bath heat block. Equal amounts of protein lysates were separated by sodium dodecyl sulfate-polyacrylamide gel (SDS-PAGE) electrophoresis at 100 V for 1 h. The protein was then transferred onto a polyvinylidene fluoride (PVDF) membrane for an hour and 30 min then blocked in a 5% milk solution [carnation powdered milk, 1× tri-buffered saline with Tween 20 (TBST)] for an hour. The membrane was incubated with FOXO3 (Cell Signaling, Danvers, MA, Cat: 75D8), or GAPDH (Santa Cruz Biotechnology, Dallas, TX, cat: G-9) antibodies overnight at 4 °C then washed for 20 min with TBST in 5-min intervals. The blot was incubated with secondary antibodies. The membrane was washed again for 20 min in 5-min intervals and allowed to develop using SuperSignal West Dura Extended Duration Substrate luminol solution for 5 min. The BioRad ChemDoc XRS+ molecular imager was used to detect light emitted from protein—containing complexes. Western blot SCN files from the (BioRad ChemDoc XRS+) were analyzed using NIH Image J.

**Fig. 5:**
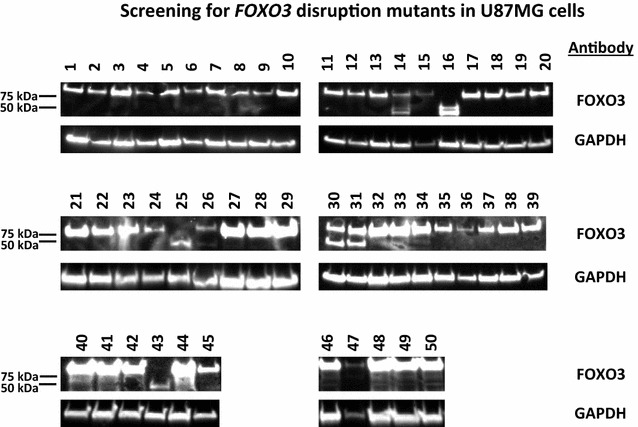
Western blot screening for putative FOXO3 truncation mutants. Total protein lysates were prepared from 50 putative *FOXO3* mutant-containing cells and were examined by western blot analyses; employed antibodies are indicated. Wild-type FOXO3 was approximately 80 kDa, whereas, mutant FOXO3 protein was approximately 45 kDa. This second proof of principle screen examined 50 potential *FOXO3* mutant clones. Three of these were homozygous mutant (16, 25 and 43), three appeared to be heterozygous (14, 30 and 31) and 44 were wild-type. The homozygous mutants were confirmed by Sanger sequencing

Western blot analysis showed that FOXO3 truncation mutant protein was much smaller than the wild-type FOXO3, roughly 45 kDa (kilo Dalton) versus the full-length 80 kDa, Fig. 4. In our first attempt to isolate *FOXO3* disruption mutants, we screened 77 putative clones and obtained four homozygous mutants based on western blot analysis, Fig. 4. Therefore, 5% of the screened isolates were homozygous mutant. In a repeat proof-of-principle experiment, we found that out of 50 screened putative mutants, three had only the truncated form of FOXO3 as evidenced by the 45 kDa band (6% homozygous mutation rate), Fig. 5. Three samples had both the truncated form of FOXO3 and the full-length FOXO3 protein (two bands that were 45 and 80 kDa), Fig. 5. 44 out of the 50 screened samples had full-length FOXO3 protein (only 80 kDa band), Fig. 5. Therefore, even with selection, there was a low mutation rate (5–6%). This was not surprising given the DNA repair deficiency found in U87MG cells [33, 34]. We also screened for FOXO3 truncation mutants in two additional cell lines: breast cancer BT549 and human kidney HEK 293. Out of 56 screened isolates in BT549 cells, five were homozygous mutant (approximately 9%), Table 3. We found that only one of 77 putative mutants screened in HEK 293 background was homozygous for *FOXO3* truncation, Table 3. Therefore the mutant frequency was between 1.3 and 9% in the tested cell lines.

**Table 3:** CRISPR Cas9 mutation frequencies in mammalian cell lines

Cell line	G418-resistant isolates screened by western blot analysis	Number of homozygous truncation mutants	Homozygous mutation frequency (%)
U87MG Trial 1	77	4^a^	5
U87MG Trial 2	50	3^a^	6
BT549	56	5	9
HEK293	77	1	1.3

^a^These mutants are shown in western blot analyses found in Figs. 4, 5

In our experiments, we were able to screen for a change in protein size. Other applications of this protocol could use similar screening techniques when the mutation frequency is low. Attachment of a GFP fusion to a gene of interest could be screened by western blot analysis, microscopy or flow cytometric analysis. Alternatively, genes could be deleted, leading to a loss of a protein of interest in western blot analysis. The ability to select mutants with neomycin and then screen using western blot analysis (or other technique) greatly facilitates the isolation of mutants.

## Genotyping CRISPR Cas9 mutants

All putative homozygous *FOXO3* disruption mutants that were identified by western blot analyses (Figs. 4, 5, and Table 3) were further confirmed using PCR and were later confirmed by Sanger sequencing. DNA was isolated from 1 million cells from each clone. The cells were removed from plates using trypsin and centrifuged at 700×*g* for 5 min. Cells were re-suspended in 500 μL of Buffer (10 mM Tris 7.4, 10 mM NaCl, 25 mM EDTA, 1% SDS). 25 μL of Proteinase K (50 U/mL stock, catalog: 03115828001, Roche, Mannheim, Germany) was added to each sample followed by an 18 h incubation at 37 °C. Next, the sample was extracted with an equal volume of phenol, followed by centrifugation at top speed in phase lock tubes (Quanta Biosciences, Beverley, MA, catalog: 2302820). DNA was precipitated from the supernatant by adding 3 μL of 20 mg/mL glycogen, 50 μL of sodium acetate and 1 mL of 100% ethanol (stored at − 80 °C over-night followed by 20 min of centrifugation at 17,000×*g*). The DNA pellets were washed with 70% ethanol, dried and re-suspended in sterile water. PCR was performed to identify clones that had *NPTII* integration into the *FOXO3* locus using the primers in Table 4. The forward primer for PCR was approximately sixty bases upstream of the *FOXO3* fragment found in Arm 1 of the *FOXO3* donor vector. Therefore, the primer sequences employed for mutant detection by PCR were absent from the donor vector and were only found in chromosomal *FOXO3*. The reverse primer was part of the *NPTII* cassette used to disrupt *FOXO3*. Most of the negative samples were positive for intact vector integration (data not shown). The PCR products (employed for detecting *FOXO3* disruption) were column purified using Qiagen PCR purification system (Hilden, Germany) and eluted with sterile water. The purified PCR product was quantified with a Nanodrop spectrophotometer. Purified PCR products were analyzed by Sanger sequencing; a primer containing *FOXO3* chromosomal sequences (that were not present in the donor vector) was utilized, Table 4. Sanger sequencing confirmed that the homozygous *FOXO3* mutants identified by western blot analyses had the designed gene disruptions.

**Table 4:** Primers utilized to detect and sequence *FOXO3* gene disruption

Primer name	Sequence
*FOXO3* F (for detection of disruption)	5′-GTGCTTCAGGATCGCTTCA-3′
Neo cassette R (for detection of disruption)	5′-TGCATGCTTTGCATACTTCTG-3′
*FOXO3* seq. (for sequencing disruption mutants)	5′-CTCGGTTTTGGACCATTCTG-3′

## Discussion

CRISPR Cas9 technology is an emergent genome editing tool. Here, we describe a protocol to disrupt the *FOXO3* gene in mammalian cells using a neomycin cassette. To decrease off-target effects, we employed 2 guide RNAs, a mutant Cas9D10A nickase and a *FOXO3* donor vector that was constructed by Gibson assembly (to enable the selection of mutants with G418). Selected mutants were validated by PCR, Sanger sequencing and western blot analysis. This protocol could be adapted to readily disrupt or modify genes of interest in order to alter the genetic background of mammalian cell lines in a directed manner. The ability to select for the disruption of genes using neomycin resistance accelerates mutant isolation, especially when mutation frequencies are low or when mutations are deleterious to cells.

Many cancer cell lines including U87MGs have deficient DNA repair [33, 34]. Homozygous mutation frequencies varied depending on the cell line as seen in Table 3. We found that even with selection, 5–6% of screened putative mutants were homozygous for *FOXO3* disruption in U87MG cells (Figs. 4, 5, Table 3). BT549 breast cancer cells had the highest efficiency of 9%. HEK 293 cells had the lowest homozygous mutation frequency of only 1.3% (Table 3), whereas many heterozygous mutants were obtained for this cell line (8 out of 77 screened by western blot, data not shown). Similar homology directed repair (HDR) frequencies were observed in HEK 293 backgrounds (0.2–1.5%) with the Cas9 D10A nickase in previous studies [24, 40, 41]. HDR frequencies using CRISPR Cas9 vary depending on the cell line, enzymes utilized (Cas9 D10A versus wild-type Cas9), transfection protocols employed, specific guide RNAs employed (including PAM sequence variances) and the specific locus being mutated [23, 27, 29, 40]. It was surprising that U87MG and BT549 cell lines had higher gene disruption frequencies than HEK 293 cells. Perhaps NHEJ more efficiently resolved the Cas9-derived nicks in HEK 293 cells, leading to lower homology directed repair in this setting. NHEJ frequencies were found to be higher than HDR frequencies in 293 backgrounds (50–60% compared to 1%, respectively) [40]. In addition, BT549 and U87MG cells harbor null mutations in the tumor suppressor *PTEN*, which impacts DNA repair via numerous mechanisms in a context-dependent manner [33, 43–46]. Loss of *PTEN* hinders DNA repair, which may shift double strand break resolution to favor HDR over NHEJ in U87MG and BT549 cell lines [33].

## Conclusions

We describe a CRISPR Cas9 genome editing protocol for mammalian cell lines by constructing and employing a custom donor vector that contains a neomycin resistance cassette. We provide a detailed, step-by-step protocol for donor vector design and construction using the pcDNA3 vector [47]. Custom donor vectors can be difficult to clone and expensive to purchase. We provide a simple, efficient protocol to obtain custom donor vectors from the common pcDNA3 mammalian expression vector. We also provide step-by-step instructions on how to select mutants and isolate clones. This protocol will allow researchers to overcome the barrier of low mutation efficiency commonly found in mammalian cell lines. Importantly, researchers can employ this protocol to build custom donor vectors in order to study novel gene functions and/or examine the localization of tagged proteins using endogenous expression levels.

## Additional file

**Additional file 1: Figure S1.** *FOXO3* donor vector complete sequence. The complete sequence of *FOXO3* donor vector is provided. *FOXO3* Arm 1 and Arm 2 are indicated. *NPTII* (neomycin resistance cassette) is indicated.

## Abbreviations

CRISPR: clustered regularly interspaced short palindromic repeats
Cas9: CRISPR associated sequence 9
NHEJ: non homologous end joining
HR: homologous recombination DNA repair
*NPTII*: *Neomycin Phosphotransferase II*
FOXO3: forkhead box O3
G418: geneticin
PCR: polymerase chain reaction
U87MG: Uppsala 87 Malignant Glioma
crRNA: CRISPR RNA
tracrRNA: trans activating CRISPR RNA
gRNA: guide RNA (for heterologous systems)
RuvC: an endonuclease domain named for an *E. coli* protein involved in DNA repair
HNH: an endonuclease domain named for characteristic histidine and asparagine residues
GFP: green fluorescent protein
HAP1: near-haploid human cell line
U2OS: human bone osteosarcoma epithelial cells
CIP: calf intestine phosphatase
*E. coli*: *Escherichia coli*
ATCC: American Type Culture Collection
MEM: minimal essential media
FBS: fetal bovine serum
Pen/strep: penicillin/streptomycin
kDa: kilo Dalton
IBC: Institutional Biosafety Committee
