# Correction to: A protocol for custom CRISPR Cas9 donor vector construction to truncate genes in mammalian cells using pcDNA3 backbone

**DOI:** 10.1186/s12867-019-0138-7

**Published:** 2019-08-14

**Authors:** Neftali Vazquez, Lilia Sanchez, Rebecca Marks, Eduardo Martinez, Victor Fanniel, Alma Lopez, Andrea Salinas, Itzel Flores, Jesse Hirschmann, Robert Gilkerson, Erin Schuenzel, Robert Dearth, Reginald Halaby, Wendy Innis-Whitehouse, Megan Keniry

**Affiliations:** 1 Department of Biology, University of Texas-Rio Grande Valley, 1201 W. University Dr., Edinburg, TX 78539 USA; 2 Department of Biology, Montclair State University, 1 Normal Ave., Montclair, NJ 07043 USA; 3 School of Medicine, University of Texas-Rio Grande Valley, 1201 W. University Dr., Edinburg, TX 78539 USA

## Correction to: BMC Molecular Biology (2018) 19:3 10.1186/s12867-018-0105-8

The original article [1] contains three erroneous mentions of usage of a restriction enzyme—*Bst*Z17I—in the Methods section as displayed in the following sentences:

1. […] Therefore, *FOXO3* gene fragments (PCR products) had pcDNA3 sequences on the ends that corresponded to upstream and downstream sequences of the utilized restriction sites (*Dra*III for Arm1 and *Bst*Z17I for Arm2) in pcDNA3.
2. […] The intermediate vector (with *FOXO3* Arm 1) was cut with *Bst*Z17I, which is on the other side of the neomycin resistance gene in the pcDNA3 plasmid compared to *FOXO3* Arm 1.
3. […] *FOXO3* Arm 2 was amplified with the primer pair specified in Table 1, producing a product that had sequences on each end that were identical to the sequences proximal to the *Bst*Z17I site in the intermediate *FOXO3* Arm 1 vector.

As such, the above three sentences should instead have stated the correct restriction enzyme—*Bsm*I—in place of where *Bst*Z17I was mentioned in each instance.
