# Supplementary material for: A protocol for custom CRISPR Cas9 donor vector construction to truncate genes in mammalian cells using pcDNA3 backbone
Source: BMC Mol Biol. 2018 Mar 14;19:3. doi: 10.1186/s12867-018-0105-8 (PMC5853148; doi:10.1186/s12867-018-0105-8)
Supplement: Supplementary file 1 — Additional file 1: Figure S1. FOXO3 donor vector complete sequence. The complete sequence of FOXO3 donor vector is provided. FOXO3 Arm 1 and Arm 2 are indicated. NPTII (neomycin resistance cassette) is indicated. [file 12867_2018_105_MOESM1_ESM.pdf]

Complete sequence of *FOXO3* donor vector

gacggatcgggagatctcccgatcccctatggtcgactctcagtacaatctgctctgatgccgcatagttaagccagtat

ctgctccctgcttgtgtgttggaggtcgctgagtagtgcgcgagcaaaatttaagctacaacaaggcaaggcttgaccga

caattgcatgaagaatctgcttagggttaggcgttttgcgctgcttcgcgatgtacgggccagatatacgcgttgacatt

gattattgactagttattaatagtaatcaattacggggtcattagttcatagcccatatatggagttccgcgttacataa

cttacggtaaatggcccgcctggctgaccgcccaacgacccccgcccattgacgtcaataatgacgtatgttcccatagt

aacgccaatagggactttccattgacgtcaatgggtggactatttacggtaaactgcccacttggcagtacatcaagtgt

atcatatgccaagtacgccccctattgacgtcaatgacggtaaatggcccgcctggcattatgcccagtacatgacctta

tgggactttcctacttggcagtacatctacgtattagtcatcgctattaccatggtgatgcggttttggcagtacatcaa

tgggcgtggatagcggtttgactcacggggatttccaagtctccaccccattgacgtcaatgggagtttgttttggcacc

aaaatcaacgggactttccaaaatgtcgtaacaactccgccccattgacgcaaatgggcggtaggcgtgtacggtgggag

gtctatataagcagagctctctggctaactagagaacccactgcttactggcttatcgaaattaatacgactcactatag

ggagacccaagcttggtaccgagctcggatccactagtaacggccgccagtgtgctggaattctgcagatatccatcaca

ctggcggccgctcgagcatgcatctagagggccctattctatagtgtcacctaaatgctagagctcgctgatcagcctcg

actgtgccttctagttgccagccatctgttgtttgcccctcccccgtgccttccttgaccctggaaggtgccactcccac

tgtcctttcctaataaaatgaggaaattgcatcgcattgtctgagtaggtgtcattctattctggggggtggggtggggc

aggacagcaagggggaggattgggaagacaatagcaggcatgctggggatgcggtgggctctatggcttctgaggcggaa

agaaccagctggggctctagggggtatccccacgcgccctgtagcggcgcattaagcgcggcgggtgtggtggttacgcg

cagcgtgaccgctacacttgccagcgccctagcgcccgctcctttcgctttcttcccttcctttctcgccacgttcgccg

gctttccccgtcaagctctaaatcggggcatccctttagggttccgattta**GTGCTTTACGGCACCTCGACCCC**

CCGGCACAACCTGTCACTGCatagtcgattcatgcgggtccagaatgagg
gaactggcaagagctcttggtggatcatcaaccctgatgggggaagagc
ggaaaagcccccggcggcgggctgtctccatggacaatagcaacaagta
taccaagagccgtggccgcgcagccaagaagaaggcagccctgcagacag
cccccgaatcagctgacgacagtccctcccagctctccaagtggcctggc
agccccacgtcacgcagcagtgatgagctggatgcgtggacggacttccg
ttcacgcaccaattctaacgccagcacagtcagtggccgcctgtcgccca
tcatggcaagcacagagttggatgaagtccaggacgatgatgcgcct**CTC**
TCGCCCATGCTCTACAGC

Human Chromosome 6 (*FOXO3* Arm 1): 108663463-108663880; regions used for primer sequences are upper case and underlined; *FOXO3* sequences are green

<u>GGCCATCGCCCTGATAGACGG</u>tttttcgccctttgacgttggagtccacgtt

ctttaatagtggactcttgttccaaactggaacaacactcaaccctatctcggtctattcttttgatttataagggattt

tggggatttcggcctattggttaaaaaatgagctgatttaacaaaaatttaacgcgaattaattctgtggaatgtgtgtc

agttagggtgtggaaagtccccaggctccccaggcaggcagaagtatgcaaagcatgcatctcaattagtcagcaaccag

gtgtggaaagtccccaggctccccagcaggcagaagtatgcaaagcatgcatctcaattagtcagcaaccatagtcccgc

ccctaactccgcccatcccgcccctaactccgcccagttccgcccattctccgccccatggctgactaattttttttatt

tatgcagaggccgaggccgcctctgcctctgagctattccagaagtagtgaggaggcttttttggaggcctaggcttttg

caaaaagctcccgggagcttgtatatccattttcggatctgatcaagagacaggatgaggatcgtttcgcatgattgaac

aagatggattgcacgcaggttctccggccgcttgggtggagaggctattcggctatgactgggcacaacagacaatcggc

tgctctgatgccgccgtgttccggctgtcagcgcaggggcgcccggttctttttgtcaagaccgacctgtccggtgccct

gaatgaactgcaggacgaggcagcgcggctatcgtggctggccacgacgggcgttccttgcgcagctgtgctcgacgttg

tcactgaagcgggaagggactggctgctattgggcgaagtgccggggcaggatctcctgtcatctcaccttgctcctgcc

gagaaagtatccatcatggctgatgcaatgcggcggctgcatacgcttgatccggctacctgcccattcgaccaccaagc

Neomycin resistance cassette (*NPTII*) in red

gaaacatcgcatcgagcgagcacgtactcggatggaagccggtcttgtcgatcaggatgatctggacgaagagcatcagg

ggctcgcgccagccgaactgttcgccaggctcaaggcgcgcatgcccgacggcgaggatctcgtcgtgacccatggcgat

gcctgcttgccgaatatcatggtggaaaatggccgcttttctggattcatcgactgtggccggctgggtgtggcggaccg

ctatcaggacatagcgttggctacccgtgatattgctgaagagcttggcggcgaatgggctgaccgcttcctcgtgcttt

acggtatcgccgctcccgattcgcagcgcatcgccttctatcgccttcttgacgagttcttctgagcgggactctggggt

tcgaaatgaccgaccaagcgacgcccaacctgccatcacgagatttcgattccaccgccgccttctatgaaaggttgggc

ttcggaatcgttttccgggacgccggctggatgatcctccagcgcggggatctcatgctggagttcttcgcccaccccaa

cttgtttattgcagcttataatggttacaaataaagcaatagcatcacaaatttca**<u>CAAATAAAGCATTTTTTTCACT</u>**

<u>CGGTGGAACTGCCACGGCTG</u>actgatatggcaggcaccatgaatctgaat
gatgggctgactgaaaacctcatggacgacctgctggataacatcacgct
cccgccatcccagccatcgcccactggggactcatgcagcggagctcta
gcttcccgtataccaccaagggctcgggcctgggctccccaaccagctcc
tttaacagcacggtgttcggaccttcatctctgaactccctacgccagtc
tcccatgcagaccatccaagagaacaagccagctaccttctcttccatgt
cacactatggtaaccagacactccaggacctgctcacttcggactcactt
agccacagcgatgtcatgatgacacagtcggaccccttgatgtctcaggc
cagcaccgctgtgtctgcccagaattcccgccggaacgtgatgcttcgca
atgatccgatgatgtcctttgctgcccagcctaaccagggaagtttggtc

Human Chromosome 6 (*FOXO3* Arm 2): 108663921-108664670; regions used for primer sequences are upper case and underlined; *FOXO3* sequences are green; the guide RNA sequence is in grey

aatcagaacttgctccaccaccagcaccaaacccagggcgctcttggtgg
cagccgtgccttgtcgaattctgtcagcaacatgggcttgagtgagtcca
gcagccttgggtcagccaaacaccagcagcagtctcctgtcagccagtct
atgcaaaccctctcggactctctctcaggctcctccttgtactcaactag
tgcaaacctgcccgtcatgggccatgagaaGTTCCCCAGCGACTTGGACC

TAGTTGTGGTTTGTCCAAACTCatcaatgtatcttatcatgtctgtataccgtcgacctctagctagagcttggcg

taatcatggtcatagctgtttcctgtgtgaaattgttatccgctcacaattccacacaacatacgagccggaagcataaa

gtgtaaagcctggggtgcctaatgagtgagctaactcacattaattgcgttgcgctcactgcccgctttccagtcgggaa

acctgtcgtgccagctgcattaatgaatcggccaacgcgcggggagaggcggtttgcgtattgggcgctcttccgcttcc

tcgctcactgactcgctgcgctcggtcgttcggctgcggcgagcggtatcagctcactcaaaggcggtaatacggttatc

cacagaatcaggggataacgcaggaaagaacatgtgagcaaaaggccagcaaaaggccaggaaccgtaaaaaggccgcgt

tgctggcgtttttccataggctccgcccccctgacgagcatcacaaaaatcgacgctcaagtcagaggtggcgaaacccg

acaggactataaagataccaggcgtttccccctggaagctccctcgtgcgctctcctgttccgaccctgccgcttaccgg

atacctgtccgcctttctcccttcgggaagcgtggcgctttctcaatgctcacgctgtaggtatctcagttcggtgtagg

tcgttcgctccaagctgggctgtgtgcacgaaccccccgttcagcccgaccgctgcgccttatccggtaactatcgtctt

gagtccaacccggtaagacacgacttatcgccactggcagcagccactggtaacaggattagcagagcgaggtatgtagg

cggtgctacagagttcttgaagtggtggcctaactacggctacactagaaggacagtatttggtatctgcgctctgctga

agccagttaccttcggaaaaagagttggtagctcttgatccggcaaacaaaccaccgctggtagcggtggtttttttgtt

tgcaagcagcagattacgcgcagaaaaaaaggatctcaagaagatcctttgatcttttctacggggtctgacgctcagtg

gaacgaaaactcacgttaagggattttggtcatgagattatcaaaaaggatcttcacctagatccttttaaattaaaaat

gaagttttaaatcaatctaaagtatatatgagtaaacttggtctgacagttaccaatgcttaatcagtgaggcacctatc

tcagcgatctgtctatttcgttcatccatagttgcctgactccccgtcgtgtagataactacgatacgggagggcttacc

atctggccccagtgctgcaatgataccgcgagacccacgctcaccggctccagatttatcagcaataaaccagccagccg

gaagggccgagcgcagaagtggtcctgcaactttatccgcctccatccagtctattaattgttgccgggaagctagagta

agtagttcgccagttaatagtttgcgcaacgttgttgccattgctacaggcatcgtggtgtcacgctcgtcgtttggtat

ggcttcattcagctccggttcccaacgatcaaggcgagttacatgatcccccatgttgtgcaaaaaagcggttagctcct

tcggtcctccgatcgttgtcagaagtaagttggccgcagtgttatcactcatggttatggcagcactgcataattctctt

actgtcatgccatccgtaagatgcttttctgtgactggtgagtactcaaccaagtcattctgagaatagtgtatgcggcg

accgagttgctcttgcccggcgtcaatacgggataataccgcgccacatagcagaactttaaaagtgctcatcattggaa

aacgttcttcggggcgaaaactctcaaggatcttaccgctgttgagatccagttcgatgtaacccactcgtgcacccaac

tgatcttcagcatcttttactttcaccagcgtttctgggtgagcaaaaacaggaaggcaaaatgccgcaaaaaagggaat

aagggcgacacggaaatgttgaatactcatactcttcctttttcaatattattgaagcatttatcagggttattgtctca

tgagcggatacatatttgaatgtatttagaaaaataaacaaataggggttccgcgcacatttccccgaaaagtgccacct

gacgtc
